# Learning Teach-Back in clinical practice: a qualitative study of healthcare professionals

**DOI:** 10.1186/s12909-026-10315-6

**Published:** 2026-09-07

**Authors:** Emelie Mälstam, Charlotte Ytterberg, Maria Flink, Malin Tistad, Terese Stenfors, Lena von Koch, Sebastian Lindblom

**Affiliations:** 1 Faculty of Health and Occupational Studies, University of Gävle, Gävle, Sweden; 2 Department of Neurobiology, Care Sciences and Society, Karolinska Institutet, Stockholm, Sweden; 3 Theme Women’s Health and Allied Health Professionals, Karolinska University Hospital, Huddinge, Sweden; 4 Research and Development Unit for Older Persons, Region Stockholm, Stockholm, Sweden; 5 School of Health and Welfare, Dalarna University, Falun, Sweden; 6 Department of Learning, Informatics, Management and Ethics, Karolinska Institutet, Stockholm, Sweden; 7 Theme Heart & Vascular and Neuro, Karolinska University Hospital, Stockholm, Sweden; 8 Department of Health Sciences, Innovation and Design, Mälardalen University, Västerås, Sweden

**Keywords:** Workplace learning, Feedback, Health communication, Health literacy, Stroke prevention, Organizational learning, Practice-based learning, Qualitative method

## Abstract

**Background:**

Teach-Back is a widely recommended communication method to support patient understanding, yet little is known about how healthcare professionals learn Teach-Back in everyday clinical practice. The study aimed to explore how healthcare professionals learn Teach Back within an acute hospital setting, and how this learning is experienced and enacted in everyday clinical practice.

**Methods:**

This qualitative study invited staff in two healthcare settings that had partaken in a Teach-Back method training program. Participants included 18 healthcare professionals and four first-line managers. Data were generated through interviews conducted in the beginning and after the training program, and through observations of clinical encounters when applying the Teach-Back method. Data were analyzed using reflexive thematic analysis.

**Results:**

Learning Teach-Back was identified as a multilevel workplace learning process that developed through interactions between individual experiences, interpersonal learning processes, organizational engagement, and workplace conditions. This was reflected in four interrelated themes: (1) Individual level: Learning by doing and experiencing Teach-Back: authentic Teach-Back encounters with patients were critical for skill development but could trigger insecurity when Teach-Back was experienced as unnatural. Emotional responses, discouragement and “aha” moments, shaped whether healthcare professionals used Teach-Back or not; (2) Interpersonal level: Learning through feedback and shared meaning: recurring peer reflection and supportive feedback were crucial for learning Teach-Back, yet often lacking; (3) Organizational level: Engaging the organization to promote learning: early adopters, ambassadors, leadership support, and interprofessional communication were decisive for learning Teach-Back; and (4) Workplace conditions: Making space for learning in a pressured work environment: staff shortages, turnover, and high workload constrained opportunities for doing and engaging in reflection and feedback about Teach-Back.

**Conclusions:**

Learning Teach-Back can be understood as a multilevel workplace learning process that develops through opportunities for practice, reflection, and collegial feedback in everyday clinical work. Supporting Teach-Back learning requires workplace learning approaches that foster shared meaning across professional teams, organizational engagement, and protected space for learning.

**Trial registration:**

ClinicalTrials.gov ID: NCT02925871. Registration date: first submitted 8 April 2016; first posted 6 October 2016.

**Supplementary Information:**

The online version contains supplementary material available at https://doi.org/10.1186/s12909-026-10315-6.

## Background

Modern healthcare increasingly expects patients to take an active role in managing their care [1, 2]. This often involves receiving and processing substantial amounts of information, for example at hospital discharge, which includes guidance on treatments, medication management, coordinated follow-ups across providers, and lifestyle adjustments to address chronic conditions [3]. The ability to understand and apply such information is critical for safe recovery [4], and secondary prevention of non-communicable diseases such as stroke [5]. Yet, patients are often vulnerable at the point of discharge [6] and exposed to time-pressured communication with healthcare professionals (HCPs) [3]. The demands can be overwhelming and may exceed patients’ capacity to engage meaningfully in their care [3, 6]. This highlights the need for healthcare approaches, led by HCPs, that not only facilitate patients’ understanding of medical information but also support them in applying it in their everyday life [7].

The capacity to access, understand, and apply health information constitutes health literacy [8], a fundamental determinant of health outcomes and health management [9]. Patients with limited health literacy are more likely to experience difficulties in understanding information and therefore require support to retain and recall it [10]. Supporting patient understanding is not solely dependent on the content of information provided but also on how HCPs communicate and adapt information to individual patients’ needs and circumstances. This places considerable demands on HCPs’ communicative competence, particularly in complex situations such as discharge planning and care transitions.

The Teach-Back (TB) method is a strategy to increase patient understanding and adherence to treatment and medication management [11]. TB involves the use of plain language; the delivery of information in segmented “chunks”; and following each segment the patient is respectfully invited to restate the information in their own words, thereby enabling HCPs to “check” for patient understanding [12]. Evidence shows that the clinical application of TB improves patients’ understanding and recall [13], promotes adherence to health behaviors, leads to positive health outcomes [14, 15], and reduces hospital readmissions [16]. Consequently, TB has been increasingly promoted as a health literacy-responsive communication practice, particularly in contexts where patients receive complex information and are expected to manage aspects of their care independently after discharge [11, 17].

Despite the importance of improving healthcare professionals’ (HCPs) communicative competence to enhance patient understanding [18], the use of TB in healthcare remains inconsistent [15, 19, 20]. Reported barriers include HCPs’ lack of confidence, uncertainty regarding how to use the method, insufficient opportunities for practice, and limited training in health literacy-responsive communication [21]. While educational interventions have demonstrated improvements in TB-related knowledge and skills among HCPs [22–25], less attention has been directed towards how professionals learn TB in everyday clinical practice. Workplace learning recognizes that HCPs develop new knowledge and skills not only through formal education but also through engagement in everyday clinical work and interactions with colleagues and patients [26, 27], and is shaped by the interaction between individual engagement and the clinical environment [26]. However, little is known about how HCPs learn TB through everyday clinical practice or how this learning is experienced within acute hospital care. Therefore, the aim of this study was to explore how HCPs learn TB within an acute hospital setting, and how this learning is experienced and enacted in everyday clinical practice.

## Methods

### Study design

This qualitative study was a secondary analysis of data generated from interviews and observations with HCPs and first line managers, conducted within a feasibility study of a co-designed care transition support intervention for people with stroke, “the Missing Link project” [28–30]. The multicomponent care transition support included components such as “what-matters-to-me” dialogue, a coordinated rehabilitation plan, and a bridged e-meeting. TB was the key component, integrated as the primary communication method across all intervention components to support person-centered communication during discharge preparation and early post-discharge contacts.

Ethical approval was obtained (Ref. 2015/1923-31/2, 2019–04219, 2021–02274), and the feasibility study was registered at ClinicalTrials.gov (NCT02925871). Registration date: first submitted 8 April 2016; first posted 6 October 2016.

### Setting and context

Data were collected between September 2021 and February 2022 within a previously described care trajectory [31], involving an acute stroke unit, a geriatric ward at a hospital, and two corresponding neurorehabilitation teams in primary healthcare in Stockholm, Sweden. HCPs at the hospital wards form multidisciplinary teams including physicians, nurses, assistant nurses, occupational therapists, physiotherapists, and speech language therapists. While these professionals work together on the wards, they can be employed within separate organizational hospital units with different managers. The corresponding neurorehabilitation teams were also different HCPs in primary healthcare.

### Participants and sampling

In total, 46 individuals were invited to participate, including HCPs working in the acute stroke unit (10 physicians and 8 allied health professionals) and the geriatric ward (4 physicians and 7 allied health professionals), along with staff from two corresponding neurorehabilitation teams (8 in total), were invited to participate. First-line managers were also invited (5 from the stroke unit, 2 from geriatrics, and 2 from the neurorehabilitation teams). All participants received both oral and written information about the study. Those who agreed to take part provided written informed consent prior to enrolment.

In total, 22 interviews were conducted with HCPs (*n* = 18), and first-line managers (*n* = 4). Nine interviews were carried out at the beginning of the study: six individual interviews (four first-line managers and two HCPs) and three group interviews with HCPs from the acute stroke unit, the geriatric ward, and one neurorehabilitation team. After completion of the HCPs training, an additional 14 interviews were conducted, comprising nine individual interviews with HCPs from the acute stroke unit, and five with HCPs from the geriatric ward. The duration of the interviews varied between 20 and 55 min, and observations between 5 and 20 min.

A total of 11 patient–provider encounters were observed, involving 10 of the participating HCPs. The observed encounters covered typical one-to-one patient-provider situations in which TB was expected to be used. This included occupational therapy and physiotherapy patient encounters on the wards, joint sessions with multiple HCPs, physician-led discharge meetings, and one bridged e-meeting involving HCPs at the geriatric ward and one neurorehabilitation team. Table 1 shows participants’ characteristics.

**Table 1:** Characteristics of study participants

	Healthcare professionals (*n* = 18)	First-line managers (*n* = 4)
Characteristics		
Age, mean (range) years	39 (27–64)	50 (45–55)
Biological sex, n (male/female)	1 / 17	0 / 4
Profession, n		
* Occupational therapist*	8	1
* Physiotherapist*	4	1
* Physician*	4	1
* Speech and language therapist*	2	1
Years in profession, mean (range)	11 (0.5–32)	21 (15–27)
Years at current workplace, mean (range)	6 (0.5–24)	10 (1–27)

### Training program

The training program, inspired by a TB website [32], was developed and delivered by three instructors (second, third and last authors). It included an educational video on the purpose and practical use of TB, followed by role-play and clinical scenarios with opportunities to reflect and receive feedback on TB performance. Participants also discussed how TB could be applied during different stages of a hospital stay and structured opportunities between sessions for reflection on HCPs experiences of using TB in practice.

**Fig. 1:**
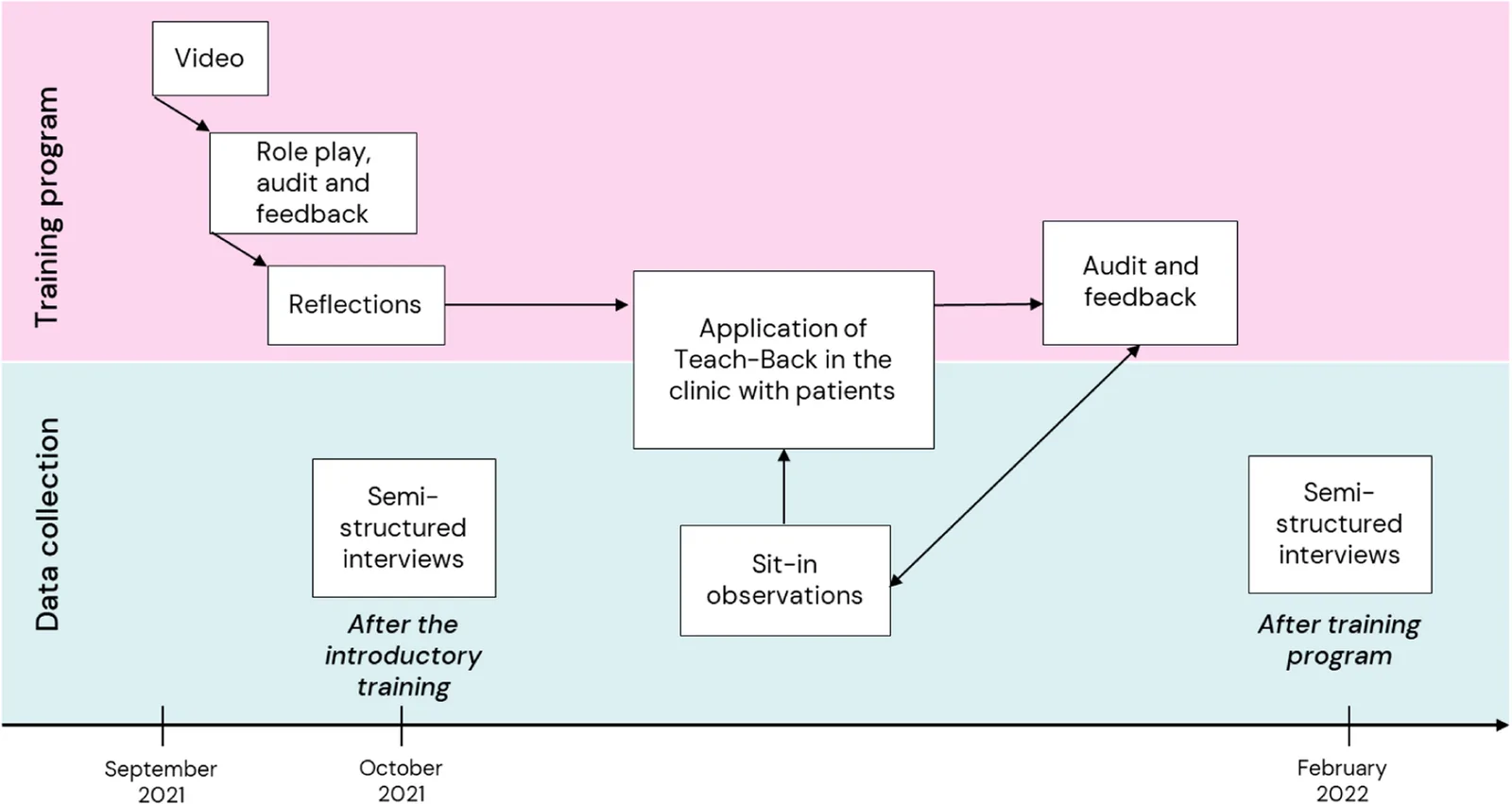
An overview of learning activities, data collection methods, and time points

After the introductory training, a central element of the training program was that participants subsequently used TB in their clinical practice, with opportunities for audit and feedback based on observed encounters. Participants were encouraged to use Teach-Back during clinical encounters related to the care transition intervention, including discharge preparation, rehabilitation planning, patient education, and follow-up communication. The purpose was to support patients’ understanding of information necessary for a care transition from hospital to home and subsequent self-management. Figure 1 provides an overview of the learning activities and data collection.

As physician staffing altered during the study period, ongoing TB education was offered. All HCPs also received pocket reminders summarizing key TB components.

### Data collection

Data were collected by the last author through semi-structured individual and group interviews with HCPs and first-line managers, and through sit-in observations of patient–HCP encounters. Interviews were conducted on two occasions; after the introductory training, focusing on experiences of learning TB, and after the training program, focusing on learning and applying TB in clinical practice. Individual or group formats were chosen based on the participants’ preferences to minimize disruption to clinical work.

The semi-structured interview guides contained open-ended questions encouraging participants to describe experiences of learning TB, how they understood the method, how they attempted to apply its elements in patient encounters, and perceived barriers and facilitators to learning in clinical context. As the interview guides were developed for the broader feasibility study, they also included questions related to other aspects of the intervention, alongside specific questions and prompts addressing experiences of learning TB. Although the interviews had been developed for the broader feasibility study, questions specifically addressing TB learning were included in all interviews. The interview guides were developed by the research team and are provided as supplementary material (see Supplementary files 1–3).

Observations were carried out during the period in which participants were encouraged to apply TB and could receive audit and feedback. To capture naturally occurring opportunities to observe TB use, the researcher spent time on the wards and observed suitable encounters as they arose, resulting in variation in the number of observations per participant. Before observations, patients received verbal information about the researcher’s presence and purpose of observations. Observations proceeded only after oral consent was obtained. Observations focused on how HCPs used core TB principles and were documented through field notes taken during and immediately after encounters. Field notes were transcribed into full-text documents. All interviews were audio-recorded and transcribed verbatim. Transcripts and observational notes were managed in Microsoft Word and ATLAS.ti.

### Analysis

Analysis followed a six-step data-driven inductive reflexive thematic analysis (RTA) [33]. Themes were developed by identifying recurring and shared patterns of meaning relating to HCPs learning and experiences of TB. Analysis began with first author thoroughly reading transcripts to ensure familiarity with the data, as she had not conducted interviews or observations. The transcripts of the interviews and observations were subsequently coded in ATLAS.ti.

During familiarization, the first author identified substantial material relating to participants’ experiences of learning, using and reflecting on TB. Recurring accounts of TB learning were identified across professions, interview occasions, and observational data, indicating sufficient richness and variation to support thematic development.

Observational data were coded and analyzed alongside interview data and contributed both to the development and refinement of themes, and allowing examination of how TB was practiced and to relate observed behaviors with participants’ accounts.

Codes and potential themes were iteratively reviewed in regular meetings among the first and the two last authors, and at a later stage all co-authors, to challenge and enrich interpretations, reflecting the originators’ view that inductive approaches remain interpretative rather than objective [34]. The Reflexive Thematic Analysis Reporting Guidelines (RTARG) [35] informed the analysis and writing process (see Supplementary file 4).

Consistent with an interpretivist and constructivist stance, we acknowledge that the results were shaped through our active engagement with the data. Interpretations were informed by our diverse disciplinary backgrounds in health service practice and research, health communication and pedagogy. To enhance analytic transparency, key decisions were documented in reflexive journaling and critically discussed within the research team to surface assumptions, consider alternative interpretations, and maintain focus on reflexivity rather than consensus.

## Results

### Learning Teach-Back as a multilevel workplace learning process

Learning TB unfolded as a dynamic workplace learning process, which emerged through interactions between individual experiences, interpersonal learning processes, organizational engagement, and workplace conditions that together shaped opportunities for learning during everyday clinical practice. These interrelated dimensions were evident throughout clinical encounters associated with the care transition intervention, including discharge preparation, rehabilitation planning, patient education and follow-up communication. The findings are presented in four interrelated themes that illustrate these dimensions of workplace learning: (1) Individual level: Learning by doing and experiencing TB (2) Interpersonal level: Learning through feedback and shared meaning, (3) Organizational level: Engaging the organization to promote learning, and (4) Workplace conditions: Making space for learning in a pressured work environment.

### Individual level: learning by doing and experiencing Teach-Back

Learning TB was described as facilitated by hands-on doing and experiencing the method in clinical practice, expressed as *“…the more opportunities there are*,* the more ways you find to express yourself around this. Have they (patients) understood? I think it’s a matter of getting the opportunity for training. It´s something you can’t fully theorize about*,* you have to try it out”* (ID1, HCP, after training program). While trying out TB together with colleagues was important, interactions with patients during everyday clinical practice were described as even more meaningful as this provided an authentic context to understand how TB worked and had to be adapted. In several observed encounters, the use of TB required HCPs to adapt their communication in response to patients’ needs and reactions unveiling the importance of practicing and reflecting on TB with diverse groups of patients to learn the new way of communicating, especially since TB was described as differing substantially from how HCPs were used to communicating:

“We’ve found it to be an easy method to understand. You get the point and know how to do it. At the same time, we’ve felt that it clashes a bit with how we’re used to doing things” (ID2, HCP, following introductory training).

A challenge was that not all HCPs continued to use TB throughout the study. For some, TB was experienced as unnatural, leading to feelings of discomfort, self-doubt, or insecurity. Field notes from observation portrayed this insecurity:

“HCP hesitates in their expressions and looks at their notes for support, and then asks if the patient has any questions, instead of asking the person to summarize or repeat what’s been said, to check for understanding. There’s a slightly confused atmosphere in the room, as if they haven’t really understood each other with regards to whether it’s time to end (the conversation)”*.* (ID2, HCP, Observation).

The observed insecurity was also expressed verbally by HCPs: *“I felt like I was searching for other ways to phrase things to get the information across*,* but I couldn’t find the right words in that moment”* (ID3, HCP, Observation).

While some HCPs described moments as “cold showers” when they realized they had not succeeded in their use of TB :*”It’s quite a wake-up call when you realize that “wow*,* I haven´t handled this very well”* (ID4, HCP, following introductory training), others described having “aha” realization moments of the benefits of TB. For example, when HCPs asked patients to repeat ’chunks’ of what had been said, this revealed gaps in understanding: *“When they (patients) are asked to explain what’s been said*,* that’s when you realize what you might need to explain more clearly next time”* (ID5, HCP, after training program). For some, these experiences acted as catalysts for learning, nudging HCPs to retry and engage in more training, while others described feeling discouraged.

### Interpersonal level: learning through feedback and shared meaning

The importance of feedback and shared meaning among colleagues and patients were highlighted as central to learning TB, supporting reflection on one’s own performance and the adaptation of communication in practice. One HCP elaborated on this:

*“Sometimes*,* you have the theoretical knowledge*,* but it doesn’t really matter in the moment… You think you know it. Maybe it’s better to have a little… I think*,* when you’ve tried it… that you could maybe get some feedback… ‘How did you feel about it?’ And some feedback on whether there was something you could have thought differently about. It would be valuable to have someone to discuss with. That kind of hands on in the situation afterwards…without feeling like you’re being evaluated or judged*,* but a little bit like ‘how did that feel?’ or ‘was there anything…?’ Because sometimes you get insecure”* (ID6, HCP, after training program).

Follow-up meetings after using TB were one way to give and receive feedback, yet such opportunities were often lacking. This was evident in observations, where structured opportunities for feedback and reflection were rarely present. In one observed encounter, an HCP expressed appreciation for feedback during the observation:

“Thank you very much for observing. It feels reassuring to be able to reflect and receive feedback on whether I’m doing this correctly.” (ID5, HCP, Observation).

When formal planned meetings or informal conversations about TB did occur, HCPs noted these involved sharing challenging encounters, which in turn helped them process conflicting emotions. Feedback from TB instructors was also wished for, as this was described to align with the goals of TB, as one of the HCP expressed it:

“It would be valuable to get some feedback and see what I’ve understood… whether I’ve understood it correctly. A little bit of ‘teach-back’” (ID1, HCP, after training program).

When TB was adopted by several HCPs, this was suggested to foster greater motivation for learning. In contrast, when only some were using TB, HCPs described a sense of frustration: *“I believe that if you get the entire team on board*,* it will become more motivating. Because sometimes it feels like… well*,* you get the sense that it’s just us doing this. No one else does it*,* and then you start to wonder why are we doing it?*” (ID7, HCP, after training program). Without a sense of shared meaning, TB was difficult to learn as it remained disconnected from everyday clinical practice and routines.

### Organizational level: engaging the organization to promote learning

To support learning of TB, organizational engagement and communication structures were important. Early adopters acted as key assets by legitimizing TB, raising awareness, and serving as role models that supported others’ learning. As one HCP expressed it: *“She has been in the game for a long time and could potentially help spread this within the department…You need ambassadors”* (ID8, HCP, after training program). Involvement from individuals in educational or coordinating roles, as well as newer staff members who could “push” change, was described as supporting learning, as they often were open to learn.

Leadership engagement was described to set the tone for the importance of learning TB. As one HCP said: *“I believe it needs to be initiated from the top*,* at least in the beginning*,* and that everyone feels they understand what it is*,* what it’s for*,* and their role”* (ID9, manager, after training program). Leadership also helped prioritize learning for some HCPs, while others described frustration when this was absent. HCPs stressed that although key individuals at times were engaged, communicated, and used TB, these efforts could not be sustained without broader visibility and organizational communication structures that integrated the learning of TB as a natural part of everyday practice. While some HCPs integrated TB into routine forums, like interdisciplinary meetings, others experienced a more siloed process, in which knowledge about TB remained limited to a few individuals and professional groups. One HCP said:

“If everyone is involved, you could exchange ideas and discuss how it works and how it can be made easier. Both how the method itself can be improved, that more people have thoughts about it but also that more people have thoughts on how to make it easier and more efficiently can incorporate it in the daily routine” (ID10, manager, after training program).

A lack of communication structures and awareness of TB was described as undermining learning, as it led to TB being perceived as irrelevant and optional by those not directly involved. To build a collective understanding, HCPs suggested that communication about the TB method needed to be integrated in both formal settings (e.g. staff meetings) and daily conversations among the staff.

### Workplace conditions: making space for learning in a pressured work environment

Learning TB was described as being affected by the HCPs’ work conditions. The absence of TB as an integrated part of everyday clinical practice was described as limiting opportunities for learning and subsequent use in what participants described as a pressured work environment. In one observed discharge interaction, an HCP asked: *“What should I do? Do you want me to use all parts?”* (ID1, HCP, Observation), indicating uncertainty about how to apply TB but also that TB was perceived as an additional task rather than part of the existing practice.

Workplace conditions such as sick leave, parental leave, staff shortages, high staff turnover, and frequent arrival of new or temporary staff shaped the workplace context in which HCPs attempted to make space for practicing, reflecting on, and learning TB. As one HCP put it:

“This takes more time, but it provides the patient so much more. However, I believe that time will be the main challenge when it comes to implementing it on a broader scale” (ID10, manager, after training program).

Staffing issues left little room to try out TB regularly as well as reflecting on its application among peers. HCPs therefore suggested integrating the TB method within written routines and as part of the formal introduction for new staff. However, changing work conditions made it challenging to build and maintain competence in TB:

“When I am solely responsible for 22 patients, I just don’t have the time. Period. You know what I mean? There are so many people on sick leave or home with sick kids. But if there are more of us, then absolutely you do have the time” (ID5, HCP, after training program).

## Discussion

This study explored how HCPs learn TB in everyday clinical practice in acute hospital settings. The results show that learning TB was neither a linear nor solely an individual process. Instead, learning was shaped through the interaction between individual experiences, interpersonal processes, organizational engagement, and workplace conditions. Together, these findings suggest that learning TB can be understood as a multilevel workplace learning process.

At the individual level, learning occurred through experiential engagement, “learning by doing”, and through reflection and interaction with patients, colleagues, managers and instructors. These findings align with workplace learning perspectives, which emphasize that professional learning develops through engagement in authentic clinical work rather than through formal education alone [26, 36, 37]. However, HCPs also experienced discomfort, uncertainty, and difficulty maintaining TB use over time. Similar barriers, including lack of confidence [22] and discontinuation of use [20, 21] have been reported previously. Much of the existing evidence on Teach-Back training and implementation originates from nursing research, which currently represents the most developed body of literature in this field. Published studies involving the professional groups included in the present study remain comparatively limited. Although profession-specific differences may exist, many of the learning mechanisms and communication challenges described in nursing research appear relevant across healthcare professions. By including multiple healthcare professions, the present study extends existing knowledge beyond the predominantly nursing-focused Teach-Back literature. While earlier studies showed that training can improve TB knowledge among HCPs [22–25], this study extends that work by demonstrating that individual training alone is insufficient for sustained learning and practice.

Support for TB use has often focused on reminders and prompts [38–41], which were also helpful here. Yet HCPs needed to confront their deeply rooted communication habits. Collective reflection, both formal and informal, helped normalize emotionally charged experiences and transform them into learning opportunities. Having opportunities for reflection, feedback, and shared meaning are known to support the development of professional expertise [42], competence [43], and workplace learning and appear essential for learning and enacting TB in clinical practice.

From a workplace learning perspective, organizational communication structures and the visibility of TB were also crucial for shaping opportunities for learning by fostering shared meaning, motivation for change, and HCPs ability to manage the discomfort associated with learning TB. These findings align with research highlighting the importance of feedback and peer dialogue in consolidating new practices in clinical teams [44]. In this study, however, structures for reflective practice around TB were limited.

Workplace learning was also inherently interpersonal. Limited engagement with TB across professional groups risked making it appear optional or less meaningful. This illustrates how the meaning and use of TB are co-constructed within professional communities and require shared sense-making [45]. Developing TB confidence and competence thus appears to be a dynamic, iterative process involving practice, reflection, and feedback, supported by collegial engagement and conditions that promote psychological safety.

At the organizational level, the organizational conditions further shaped learning, particularly through leadership engagement and the presence of role models, which legitimized TB, encouraged continued learning and shaped the learning environment. This is consistent with workplace learning research highlighting the importance of organizational structures that support participation, reflection, and collaboration for professional learning [46, 47]. Previous work emphasizes that leadership commitment and alignment with organizational goals are essential for learning communicative practices [48]. In contrast, lack of follow-up structures created fragmented learning opportunities across professional groups. Where TB was incorporated in existing routines (e.g., team meetings), it fostered collective ownership and reduced discomfort and resistance. Sustained learning therefore required organizational scaffolding that embedded TB into both formal procedures and informal dialogue.

The workplace conditions in which learning occurred were equally important. A recurring tension was the challenge of making space for learning in high-pressure work environment, characterized by heavy workload, staffing shortages, and rapid turnover. These conditions limited opportunities for reflection, feedback, and consistent TB use. The findings mirror broader challenges of implementing new practices in complex healthcare systems where time and resources are constrained. As emphasized in prior work [11, 19, 44, 48], sustainable learning requires not only individual effort but also organizational capacity to prioritize and protect time for TB-related learning.

Taken together, the findings demonstrate that learning TB is best understood as a multilevel workplace learning process. Rather than representing external barriers or facilitators, organizational engagement and workplace conditions constituted integral components of learning by creating, and/or constraining opportunities for practice, feedback, and continued development of TB in everyday clinical work. This perspective highlights that learning communicative practices cannot rely solely on individual skill acquisition but depends on the dynamic interaction between individual experiences and engagement, interpersonal processes, organizational engagement, and workplace conditions [49].

### Strengths and limitations

A key strength of this study is the use of multiple qualitative data sources, which enabled a rich, situated understanding of how HCPs learned TB in acute healthcare settings. The longitudinal design captured experiences at two stages of the training program, following the introductory TB training and after completion of the training program, illustrating TB learning as a dynamic, context-bound process. The use of RTA [33] further supported an in-depth exploration of learning as a multilevel construct.

Participant characteristics, including age and professional experience, were reported to provide contextual information about the sample and to support readers in assessing the transferability of the findings to similar multidisciplinary healthcare settings.

Although data collection procedures aimed to support open sharing, the last author’s dual role as TB instructor and data collector may have influenced participants’ willingness to express uncertainty or criticism. However, this dual role may also have facilitated access, rapport and more in-depth accounts. The feasibility-study context also presents potential limitations, as time-constraints may have influenced participants’ behavior during observations or contributed to perception of TB learning as temporary. Despite these limitations of conducting the study within the context of the feasibility study, the results remain relevant, resonating with existing literature [22–25], and extending understanding of how TB learning is shaped by different conditions among HCPs in acute healthcare settings.

A further strength lies in the research team’s iterative and reflexive analytic engagement, including journaling and critical discussions of assumptions and analytic choices [34, 50]. For example, early analyses considered organizing results by professional roles and organizational belongings; through reflexive dialogue, we instead developed themes that captured cross-professional learning processes. By offering methodological transparency and contextual detail, we further aim to support readers in assessing the transferability of our interpretations to other multidisciplinary healthcare environments [50].

## Conclusions

Learning TB can be understood as a multilevel workplace learning process that emerges through opportunities for individual HCPs to practice the TB method in clinical work, engage in collegial feedback, and work through the uncertainty and discomfort that may arise. Learning is further shaped by a communicative culture in which shared meaning is developed across professional teams through interconnected individual, interpersonal, and organizational learning processes and workplace conditions. This highlights the need for organizational engagement, from individual HCPs to leadership, in creating communication structures that support reflection, feedback, emotional support, and ongoing learning. Equally important is a work environment that provides protected space for learning. Taken together, the findings suggest that supporting TB learning requires workplace learning approaches that integrate opportunities for practice, reflection, feedback, and organizational support into everyday clinical work. Such approaches may support the sustainable development of communication practices that strengthen patient understanding and person-centered care.

## Supplementary Information

Supplementary Material 1. Interview guide health professionals – After introductory training.

Supplementary Material 2. Interview guide health professionals – After training program.

Supplementary Material 3. Interview guide managers.

Supplementary Material 4. Reflexive Thematic Analysis Reporting Guidelines (RTARG).

## Data Availability

The datasets used and/or analyzed during the current study are available from the corresponding author on reasonable request.

## Abbreviations

HCPs: Healthcare professionals
TB: Teach–Back
RTA: Reflexive thematic analysis
RTARG: The Reflexive Thematic Analysis Reporting Guidelines
