# Supplementary material for: Learning Teach-Back in clinical practice: a qualitative study of healthcare professionals
Source: BMC Med Educ. 2026 Sep 7;26:1432. doi: 10.1186/s12909-026-10315-6 (PMC13548691; doi:10.1186/s12909-026-10315-6)
Supplement: Supplementary file 1 [file 12909_2026_10315_MOESM1_ESM.pdf]

Interview guide healthcare professionals – After introductory training

To begin with, could you describe how you perceived the Missing Link project when you first heard about it? What were your initial thoughts about the project?

- **How did you first become involved in the Missing Link project?** How did the contact take place? How did it feel at the time? Was there any resistance? If so, why?
- **Could you describe what has happened since you first became involved?** Meetings, workshops, presentations? How have you experienced the meetings, workshops, and educational sessions with researchers within the Missing Link project? What has been good/less good? What conditions did you have for participating in these meetings?
- **Could you describe how the process, i.e., the meetings, workshops, and training sessions in which you participated, prepared you to use the different components of the intervention, such as Teach-Back?** And how was it to absorb the training/information about it? Which parts did you feel most prepared for? Why? Which parts did you feel least prepared for? Why?
- **How do you perceive/experience the Teach-Back methodology? And how do you view the value of using Teach-Back?** How much consensus is there among colleagues regarding the value of using Teach-Back?
- **How do you think the structure of the Teach-Back training (number of sessions/content, etc.) supported your learning and understanding?**
- **Could the Teach-Back training have been conducted differently in any way?** If so, how?
- **In what ways have you been able to influence the development of the intervention?** If not, would you have liked to be more involved? How could this best have been achieved?
- **How has it been for you to participate in the preparations/training** (meetings, workshops, presentations)? (Conditions for participation, support from managers, colleagues, time aspects.) Barriers/facilitators? Could anything have been done differently to facilitate your participation?
- **How do you view the intervention as a whole? And the different components of the intervention?** How do you view its value? Which parts do you think are most important and least important for the patient? And why? Is there anything missing that should have been included? (Discuss the different components.)
- **How do you think the intervention differs from your usual way of working?**

- **How has it been to start using the different components of the intervention in daily practice, such as Teach-Back?** What could facilitate the use of the different components? Are there any barriers?
- **How have you received feedback about Teach-Back to understand how/whether it works?** From patients and relatives? Discussions with colleagues? Or in any other way received feedback that contributed to forming an opinion of Teach-Back as valuable/not valuable? Have you received any feedback regarding how Teach-Back is performed? From whom? What was that like? If not, would that have been desirable?
- **Is there anything you would like to add? Any other reflections and/or thoughts?**
