# Supplementary material for: Learning Teach-Back in clinical practice: a qualitative study of healthcare professionals
Source: BMC Med Educ. 2026 Sep 7;26:1432. doi: 10.1186/s12909-026-10315-6 (PMC13548691; doi:10.1186/s12909-026-10315-6)
Supplement: Supplementary file 2 [file 12909_2026_10315_MOESM2_ESM.pdf]

Interview Guide - Healthcare professionals – After training program

- **To begin with, could you describe how you perceive/experience the Missing Link project?** And your thoughts about the project? How do you view the intervention as a whole? And how do you view its value? Both for you as a physician/occupational therapist/physiotherapist/speech therapist and for the patient? What do you think can be achieved with the intervention?
- **Which parts do you think are most important and least important for the patient?** And why? Is there anything missing that should have been included? (Discuss the different components.)
- **How do you perceive/experience the Teach-Back method?** And how do you, as a physician/occupational therapist/physiotherapist/speech therapist, view the value of using Teach-Back? How much consensus is there among staff regarding Missing Link? (= the value of using Teach-Back).
  How would you describe the learning of the Teach-Back method in clinical practice?
- **How do you perceive/experience the "What Matters to Me" method?** And how do you, as an occupational therapist/physiotherapist/speech therapist, view the value of using "What Matters to Me"? How much consensus is there among staff regarding Missing Link? (= the value of using "What Matters to Me")
- **Could you describe how it has been to work with the different components of the intervention in daily practice?** (Discuss each component separately.) How has it worked practically? Could you provide an example? What has worked well? What has worked less well? What could facilitate use? Are there any barriers?
- **Do you feel that tasks related to Missing Link are currently a natural part of your work?** In what way? How did that happen? If not, do you think work related to Missing Link could become a natural part of your work? How? What would be needed? What barriers exist?
- **In what ways does the Missing Link project (the different components of the intervention) differ from your usual way of working?**
- **Did you feel sufficiently prepared to use the Teach-Back method?** Why? Which parts did you feel least prepared for? Why? Could the preparation/training have been organized differently? Could the Teach-Back training have been conducted differently in any way? If so, how?
- **How have you received feedback about Teach-Back to understand how/whether it works?** From patients and relatives? Discussions with

colleagues? Or in any other way received feedback that contributed to forming an opinion of Teach-Back as valuable/not valuable? Have you received any feedback regarding how Teach-Back is performed? From whom? What was that like? If not, would that have been desirable?

- **Do you think the staff on your ward share a common understanding and perception of what Missing Link is and its purpose?** Is it discussed at all? Why not? How could this be further developed?
- **Before a larger study, do you have any ideas on how we could encourage all professions to participate in the intervention?**
- **Will you continue using this way of working?** In what way? If not, what would be required for it to become part of your daily work?
- **Is there anything you would like to add? Any other reflections and/or thoughts?**
