# Supplementary material for: Learning Teach-Back in clinical practice: a qualitative study of healthcare professionals
Source: BMC Med Educ. 2026 Sep 7;26:1432. doi: 10.1186/s12909-026-10315-6 (PMC13548691; doi:10.1186/s12909-026-10315-6)
Supplement: Supplementary file 3 [file 12909_2026_10315_MOESM3_ESM.pdf]

Interview guide - Managers

- **Could you begin by describing your thoughts, as a manager, regarding participation in research and development projects?** Is it common for your organization to receive requests to participate? How does that process usually work? Why do organizations choose to participate or not participate?
- **Could you describe the conditions within your organization for participating in research and development projects?** Are there any barriers or facilitators? What could facilitate your participation? Funding? Policy documents? The organization's mission?
- **As a manager, what expectations do you have of researchers when it comes to collaboration on research conducted within your organization?** Before the project starts? Feedback before, during, and after the project? Other prerequisites?
- **How do you view your role as a manager when it comes to participating in research and development?** In relation to researchers, your own staff, or communication between them? When it comes to supporting staff in implementation, in relation to the rest of the organization? Or in relation to senior management?
- **Could you describe how you, as a manager, work to facilitate research and development activities within your organization?** Do you see anything that could make this easier? For example, support from other managers in the line organization? Policy documents? Financial resources? What support do you have as a manager to contribute to/facilitate research and development within your organization? What barriers exist in relation to your role as a manager?
- **Turning now to the Missing Link project. Could you describe what it was like when you first heard about the Missing Link project and what your initial thoughts about the project were?**
- **How did you/your organization first become contacted/involved in the Missing Link project?** How did the contact take place? How did it feel at that time?
- **What made you choose to involve, or not involve, yourself and your staff in the project?** Was it the topic itself, resource-related issues, or other factors?
- **How have you experienced the process since you first became involved?** How has it proceeded? Participation in meetings, workshops, presentations? Have there been any difficulties?

- **How has it been for you and your staff to participate in the preparations (meetings, workshops, presentations)?** (Conditions for participation, support from managers, colleagues, time aspects.) Barriers/facilitators? Could anything have been done differently to facilitate your/your organization's participation?
- **How would you describe the conditions for learning and enacting parts of the intervention, such as the Teach-Back method, within your unit/organization?**
- **How has the dialogue between you and your staff regarding the project been?**
- **How have you experienced the communication with the responsible researchers?** What has worked well and what has worked less well? Could anything have been done differently?
- **Is there anything you would like to add? Any other reflections and/or thoughts?**
