# Supplementary material for: Learning Teach-Back in clinical practice: a qualitative study of healthcare professionals
Source: BMC Med Educ. 2026 Sep 7;26:1432. doi: 10.1186/s12909-026-10315-6 (PMC13548691; doi:10.1186/s12909-026-10315-6)
Supplement: Supplementary file 4 [file 12909_2026_10315_MOESM4_ESM.pdf]

Supplementary material

Reflexive Thematic Analysis Reporting Guidelines (RTARG) for manuscript:

Learning and making space for Teach Back in acute hospital settings and care transitions - A qualitative study

**Advice for aspects of the research report/approach to reporting**	**Our work and reflections:**
**The Introduction**<br>NB: We prefer *Introduction* over *Literature Review* as a section heading, to capture the broader purpose of this section.	
Background and rationale	
Provide a robust context and rationale for the proposed research in the Introduction.	*We located our study within existing research on health literacy, clinical communication at discharge, and barriers to using Teach-Back (TB). We positioned ourselves in dialogue with prior work and demonstrated the need to explore how HCPs learn TB in everyday clinical practice within acute settings. We presented the research aim clearly and coherently in relation to our qualitative, interpretivist approach.*
Clearly articulate a research question – one that is methodologically coherent.	
**"Owning your perspectives"[6]**	
Include information on guiding theoretical assumptions and other (e.g., explanatory) theory informing the use of TA.	*We explicitly adopted an interpretivist and constructivist stance and described how this guided data generation, analysis and interpretation. We acknowledged researcher subjectivity as a resource, reported diverse disciplinary backgrounds, and described how reflexive journaling and team dialogue shaped the analytic process.*
Report in a way that is consistent with stated theoretical assumptions throughout.	
Evidence methodological coherence/integrity in both the research and the report.7	
Show evidence of reflexive practice.	
Write in a methodologically coherent style.	
**The Methodology**<br>NB: We prefer the theoretically-embedded term *Methodology* as a section header, over the proceduralist term *Method*.	
Participants/data items	
Describe selection of participants/data items.	*We described who was invited, who participated, professional roles, and the rationale for involving HCPs and first-line managers across acute, geriatric and primary care settings. We provided aggregate demographic information to*
Describe number of participants/data items; provide a rationale or explanation around dataset or participant group size/composition.	
Discuss characteristics of participants/data items.	

	maintain anonymity. Our justification for dataset size was grounded in qualitative sufficiency rather than saturation.
Detail ethical approval and ethical code/principles followed, participant informed consent, etc.	*We reported ethical approval from relevant Swedish authorities and stated that participants provided verbal and written informed consent. We described measures to safeguard participants identities and voluntary participation.*
Dataset generation<br>NB: We prefer the term *generation* over *collection* to capture the active role of the researcher and that data don't pre-exist research *as* data, but *become* data through research practices.	
Provide some rationale for method(s) for data generation/data item sources chosen.	*We write that semi-structured interviews and observations are appropriate for exploring experiential learning in practice contexts. We described the background and rationale of the study as a secondary analysis and that the development of interview guides, settings and timing of data generation was connected to the feasibility study, and who conducted the interviews and observations.*
Describe development and/or characteristics of data generation tool(s).	
Include details such as modality and/or setting of data generation, time frame, and other pertinent procedural information.	
Describe who conducted any interactive data generation (which author or research role), and how.	
Describe the size/scope of dataset and dataset items.	
Describe, and if relevant explain, any preparation of data for analysis.	
Data analysis	
Provide some rationale for use of RTA, and, where relevant, for combining RTA with other approaches and procedures.	*We justified RTA as fitting our aim to explore patterns of shared meaning about learning TB across a multilevel clinical context. We explained that RTA enabled an inductive, interpretive engagement with the data in the research group.*
Describe specific orientation to RTA.	*We located our analysis as inductive and primarily semantic, with attention to latent elements when relevant. We positioned themes as constructed through researcher–data interaction rather than discovered and "emerging".*
Discuss how the researcher(s) engaged with the analytic process.	*We described a six-step analytic process aligned with RTA, including familiarization, coding, iterative theme development, and reflexive team dialogue. We documented reflexive practices, including journaling and analytic discussions that challenged assumptions and supported interpretation.*

Where more than one person is involved, describe who analysed the data (author or research role).	*We detailed how the first author conducted initial coding and how the analytic team (EM, SL, LvK) collaboratively reviewed codes and theme candidates, and also discussing with the remaining co-authors in later stages in relation to finilizing the results. We stressed interpretative engagement rather than consensus checking.*
Use language to describe the process and products of RTA that is coherent with the values and assumptions of RTA.	*We used language consistent with RTA (e.g., developing, constructing, shaping themes) and avoided discovery-oriented terminology.*
**The Analysis**<br>NB: We prefer the heading *Analysis* over *Findings/Results*. *Findings* implies the researcher "found", "discovered" or "identified" pre-existing themes. *Results* is strongly associated with the outputs of statistical analysis.	
Reporting the data analysis	
Provide an overview of themes or thematic structure.<br>Ensure theme conceptualisation is appropriate to RTA, and any divergences are justified and explained.	*We presented four themes, each illustrated with data extracts and accompanied by analytic narrative. Themes were united by central organizing concepts rather than topic summaries.*
Name themes appropriately.<br>Report themes in sufficient depth and detail.<br>Use subtheme judiciously.	*Themes reflected shared meaning patterns about how HCPs learn and enact TB across individual, group and organizational levels. Subthemes were not used, as themes were multifaceted and sufficiently elaborated.*
Ensure the analytic narrative explains the meaning and significance of the data.<br>Provide an appropriate *balance* of analytic narrative and data extracts – both data extracts *and* analytic narrative matter.<br>Demonstrate coherence between analytic narrative and illustrative/evidentiary data extracts.<br>Integrate existing research and theory into the analytic narrative.	*We used some narratives to explain the meaning of the data, but did not incorporate all.*<br><br>*We did not integrate experiential and situated learning theories and research on communication practices into the results, rather kept this part for the discussion of the overarching results.*
**The Final Section – A General Discussion or "Conclusions"**<br>NB: We don't have a preference for what a final section of an RTA report is called, and it depends on the context and the focus and purpose of the study – the heading *Conclusion* may evoke a certainty that isn't appropriate; *Implications* may be useful; *Final Considerations* or *Reflections* may work, as might *General Discussion*.	
Quality, evaluation and conclusions	
Draw analytic conclusions across themes.	*We synthesised insights across themes to articulate how learning TB is a multilevel process requiring experiential practice,*

	shared meaning, supportive structures, and organizational engagement.
Discuss implications or directions for future research.	*We offered evidence-based recommendations for supporting TB learning and organizational strategies needed to sustain communicative practices.*
Use and report quality practices coherent with RTA.	*Our quality practices included reflexive journaling, iterative team dialogue, methodological transparency, and analytic integrity consistent with RTA. We did not use positivist validation tools such as triangulation or inter-coder reliability.*
Evaluate the research from a *Big Q* standpoint.	*We reflected on how context, participant characteristics, and our methodological choices shaped the knowledge produced, and framed situatedness as a strength rather than a limitation.*
Include reflections on research process and practices, including researcher reflexivity.	

References

1. Braun V and Clarke V. One size fits all? What counts as quality practice in (reflexive) thematic analysis? *Qual. Res. Psychol*. 2021; 18: 328-352.
2. Braun V and Clarke V. Conceptual and design thinking for thematic analysis. *Qual Psychol*. 2022; 9: 3-26.
3. Braun V and Clarke V. *Thematic analysis: A practical guide*. London: Sage, 2022.
4. Braun V and Clarke V. How do we solve a problem like COREQ? A critique of Tong et al.'s (2007) consolidated criteria for reporting qualitative research. Unpublished.
5. Braun V and Clarke V. Reporting guidelines for qualitative research: A values-based approach. Unpublished.
6. Elliot R, Fischer CT and Rennie DL. Evolving guidelines for publication of qualitative research studies in psychology and related fields. *Br. J. Clin. Psychol.* 1999; 38: 215-229.
7. Levitt HM, Motulsky SL, Wertz FJ, Morrow SL and Ponterotto JG. Recommendations for designing and reviewing qualitative research in psychology: Promoting methodological integrity. *Qual Psychol*. 2017; 4: 2-22.
8. Malterud K, Siersma VK and Guassora AD. Sample size in qualitative interview studies: Guided by information power. *Qual. Health Res.* 2016; 26: 1753-1760.
9. Tracy SJ. Qualitative quality: Eight "big-tent" criteria for excellent qualitative research. *Qual Inq*. 2010; 16: 837-851.
10. Braun V and Clarke V. (Mis)conceptualising themes, thematic analysis, and other problems with Fugard and Potts' (2015) sample-size tool for thematic analysis. *Int. J. Soc. Res. Methodol*. 2016; 19: 739-743.

11. Sandelowski M and Leeman J. Writing usable qualitative health research findings. *Qual. Health Res.* 2012; 22: 1404-1413.
12. Smith B and McGannon KR. Developing rigour in qualitative research: Problems and opportunities within sport and exercise psychology. *Int Rev Sport Exerc Psychol.* 2017; 11: 101-121.
13. Smith B. Generalizability in qualitative research: Misunderstandings, opportunities and recommendations for the sport and exercise sciences. *Qual. Res. Sport Exerc. Health.* 2018; 10: 137-149.
